# Dental care professionals’ awareness of oral dryness and its clinical management: a questionnaire-based study

**DOI:** 10.1186/s12903-023-03813-2

**Published:** 2024-01-08

**Authors:** Amela Fisic, Hulya Cevik Aras, Ulrica Almhöjd, Annica Almståhl

**Affiliations:** 1 Dept of Oral Health, Faculty of Odontology, Malmö University, Malmö, Sweden; 2 Dept of Oral Medicine and Pathology, Institute of Odontology, Sahlgrenska Academy, University of Gothenburg, Gothenburg, Sweden; 3 Specialist Clinic of Oral Medicine, Public Dental Care, Region Västra Götaland, Västra Götaland, Sweden; 4 Dept of Cariology, Institute of Odontology, Sahlgrenska Academy, University of Gothenburg, Gothenburg, Sweden; 5 Dept of Oral Microbiology and Immunology, Institute of Odontology, Sahlgrenska Academy, University of Gothenburg, Gothenburg, Sweden

**Keywords:** Dentists, Dental care, Dental hygienists, Dry mouth, Sunet survey, Questionnaire

## Abstract

**Background:**

Despite the high prevalence of oral dryness and awareness of its complications, there is limited research on the clinical management of patients with oral dryness in general dental care.

**Purpose:**

To (1) describe and compare awareness among dental care professionals regarding saliva functions, potential causes and complications of oral dryness, and patient management (2) Investigate if the length of professional experience influences these aspects.

**Methods:**

A digital self-administrated survey was sent to 2668 dental care professionals working in the general dental care, Public Dental Service, in Sweden. Twelve dental care professionals reviewed the questionnaire prior to its distribution. The questionnaire comprised 32 questions about patient management, awareness of saliva functions, causes and complications of oral dryness, and self-assessment queries.

**Results:**

The response rate was 18.6% (241 dentists and 257 dental hygienists). Older adults (65+) were asked more often about dry mouth (93.0%) compared to those aged 18–23 years (50.0%) and those under 18 years (24.9%). Dental hygienists encountered individuals with oral dryness more frequently (61.1%) than dentists (48.5%) (p < 0.01), and more often asked individuals in the age groups 18–23 years (p = 0.003), 24–40 years (p = 0.045), and 41–65 years (p = 0.031) about dry mouth. A higher proportion of dental hygienists (88.3%) than dentists (51.0%) had measured salivary secretion rate, (p < 0.001) and more often suggested preventive dental care 3–4 times a year, (42.5% vs. 30.5%) (p < 0.007). Dentists had a higher awareness of saliva functions, while dental hygienists had a higher awareness about causes and complications of oral dryness. Higher proportions of dentists and dental hygienists with over 10 years of professional experience had measured salivary secretion rate (69.1% vs. 95.7%) compared to their counterparts with less than 10 years of professional experience (35.9% vs. 79.5%) (p < 0.001 for both).

**Conclusion:**

Compared to dentists, dental hygienists were more attentive to patients with oral dryness as they encountered these individuals more often, asked more age-groups, suggested frequent preventive measures, and had higher awareness of the causes and complications of oral dryness. Length of professional experience could improve both the management of patients with oral dryness and awareness of its causes, particularly for dental hygienists.

## Introduction

Saliva has several important functions essential to maintaining overall health, as well as oral health [1]. It is important for oral homeostasis with numerous functions, which include lubricating soft tissues, regulating pH levels, clearing food particles, antimicrobial function, and facilitating tooth mineralization [1–3].

Oral dryness is a complex condition [1, 4–6] encompassing both the subjective sensation of dry mouth (Xerostomia) and objective evidence of reduced saliva production (hyposalivation) [1, 3, 7]. Xerostomia can only be diagnosed by assessing the subjective symptoms of the individual, whereas hyposalivation is determined by measuring the salivary flow rate [8]. Hyposalivation is diagnosed when saliva secretion rate is reduced, for unstimulated saliva to ≤ 0.1 ml/min, and for stimulated saliva to ≤ 0.7 ml/min [1, 2]. The subjective sensation of dry mouth does not always correlate with hyposalivation, which suggests a change in saliva composition that may contribute to the perception of dry mouth [1, 2, 9].

Medications are the predominant cause of oral dryness because of their side effects. Sjögren’s syndrome, radiation therapy of the head and neck region, and systemic diseases are other causes of oral dryness [1–3]. Individuals suffering from oral dryness often experience difficulties with eating, swallowing, speech, and taste alterations, as well as dental caries [1, 10, 11]. Oral dryness can significantly affect oral health, quality of life, and well-being negatively [1, 12, 13]. A careful examination including questions about dry mouth in different situations, extra- and intraoral examination and measurement of salivary flow rates aids in the diagnosis [1, 14]. There is no treatment of oral dryness, but saliva stimulating products and saliva substitutes (mouth gel or sprays) can reduce the symptoms [8]. Most products have only a short-lived effect [8, 15]. The consequences of oral dryness could be decreased with preventive measures. Dental care professionals have an important role in raising patient awareness and knowledge of oral dryness [8].

A systematic review with meta-analysis showed the prevalence of dry mouth is higher among older adults (25.3%) compared to younger adults (19.3%) [16]. In a Swedish study including adults aged 50–75 years old, the prevalence of xerostomia was 33.9% [17, 18]. A recently published study in primary health care in Sweden showed that xerostomia was a common problem among adults regardless of age, with an overall prevalence of 43.6% [19]. Oral dryness is more common among women than men [17–20].

With an increasing number of older people [21], oral dryness will likely be more frequent in the future. Exploring how dentists and dental hygienists in general dental care address oral dryness is important, but there are few studies on this subject. According to a German study, dentists were aware of the psychological and clinical consequences of dry mouth but were unsure of how to identify and treat the affected patients [22]. Similarly, another study showed a high awareness of the condition but uncertainty on how to manage and treat individuals with dry mouth [23]. Therefore, comprehensive knowledge of saliva functions and identification of patients with oral dryness is crucial to implement management strategies and individual treatment plans for such patients [10]. A Swedish interview study showed that healthcare professionals, including dentists and dental hygienists, lacked sufficient knowledge in managing patients with dry mouth [24]. However, to the best of this study’s knowledge, no studies are currently available regarding the management of patients with oral dryness by dentists and dental hygienists in Sweden.

The aims of this study were therefore (1) to describe and compare awareness among dental care professionals regarding saliva functions, potential causes and complications of oral dryness, and patient management (2) Investigate if the length of professional experience influences these aspects.

## Materials and methods

### Ethical aspects

The Swedish Ethical Review Board has approved the study (Dnr: 2021 − 01795). A letter providing information about the purpose of the study, the confidentiality of participation, and that participation was voluntary was sent to all participants. All participants consented to take part by completing and returning the questionnaire. The responses from the questionnaire were handled confidentially.

### Study design

This is a cross-sectional survey study including dental hygienists and dentists employed within the Public Dental Service in Sweden. STROBE statement checklist for cross-sectional studies were used to accomplish a comprehensive description of the study [25].

### Sample selection

The survey was distributed to all 2668 dental care professionals, including both dentists and dental hygienists, working in general dental care, Public Dental Services in five counties in Sweden: Region Skåne (n = 561), Region Stockholm (n = 856), Region Västra Götaland (n = 1029), Region Kalmar (n = 100), Region Dalarna (n = 122). The counties were selected based on convenience, as well as encompassing both cities and rural areas in Sweden. Inclusion criteria were general dentists and dental hygienists working in Public Dental Services. Exclusion criteria were dentists or dental hygienists working in the specialist dental care.

### Questionnaire

The questionnaire encompassed four sections; (1) demographics (11 questions) (2) content of undergraduate education (5 questions) (3) approaches used in clinical practice for the management of individuals with oral dryness (11 questions), (4) awareness questions of saliva functions, causes and complications associated with oral dryness (4 questions) and (5) a concluding question where all participants were asked if they wish for further education on dry mouth and oral health.

To assess the respondents’ levels of agreement regarding their awareness of saliva functions, factors that may cause oral dryness, and complications associated with oral dryness, the questionnaire utilized a 5-point scale ranging from 1 (strongly agree) to 5 (strongly disagree), with an additional option for “don’t know”. To capture the dental care professional approaches used in clinical practice for managing individuals with oral dryness, nine questions with fixed answer alternatives were used. Two questions focused on how information about oral dryness was provided, and one question evaluated the recommended treatment options. The response options for these questions included “always”, “often”, “seldom”, “never”, and “don´t know”. For the three self-evaluation questions, a 4-point scale (very good, good, satisfying, insufficient) was used for the respondents to assess their knowledge levels regarding the consequences of oral dryness, clinical management, and treatment options for individuals with oral dryness. All questions were mandatory to answer.

### Evaluation of the preliminary questionnaire

The preliminary questionnaire was distributed to four colleagues and revised according to their feedback. To further evaluate the effectiveness of the questionnaire, twelve dental care professionals (3 dental hygienists and 9 dentists), working in the Public Dental Service in Trollhättan, Sweden (Region Västra Götaland), completed the questionnaire. A few minor adjustments were made, i.e., reducing some of the response options in certain questions, as the respondents felt there were too many answer alternatives. Technical issues with the questionnaire were also corrected.

### Procedure

The authors developed a self-administered, digital survey consisting of 32 questions. The survey was designed using web-based software called Artologik software for the web (Sunet Survey). The questionnaire was distributed via email from November 2021 to November 2022 and included an information letter and a link to the survey. A research-coordinator in the included region was responsible for the distribution, except in one region where AF handled the distribution. Two reminders were sent out at two-week intervals. The questionnaire took approximately 10 min to complete.

### Statistical analysis

Descriptive statistics, including measures such as mean, standard deviation, and percentage, were used. The data was analysed through cross-tabulations, and differences in responses between the two professions were assessed using either the Chi-squared test or Fisher’s exact test. Dentists and dental hygienists were divided into two groups based on their professional experience: less than 10 years or over 10 years. This cut-off was used to facilitate statistical analysis since the groups were similar in size. For the three questions regarding the functions of saliva, causes of oral dryness, and complications associated with oral dryness, the responses were dichotomized into two options; agree *(strongly agree, agree)* or disagree *(strongly disagree, disagree, don’t know)*, since agreement is the only correct answer and indicates a high level of awareness.

For the questions related to providing information to individuals with oral dryness, the response alternatives: *always, often, rarely, never, don’t know* were dichotomized into two response options; always/often *(always, often)* and other *(rarely, never, don´t know)* since most of the respondents answered often and rarely. The question regarding treatment options was also dichotomized into the same response options since few respondents used the response options; *never*, *don´t know*. The question regarding how frequently the dental care professionals met patients with oral dryness was dichotomized into; *several times/week or more often* and *once a week or less*. A p-value of < 0.05 was regarded as statistically significant. The statistical analysis was performed using IBM SPSS statistics software version 28.0.1.1 (14).

## Results

### Participation rate and characteristics of the study participants

The response rate was 18.6% (Table 1). Twenty-four respondents (4.8%) stated that they did not work clinically (5.8% of the dentists and 3.9% of the dental hygienists) and were therefore excluded from all parts of the survey except Sect. 4. One respondent’s answers to all the questions in Sect. 3 were excluded since they reported not meeting individuals with oral dryness.

**Table 1:** Age, gender, years, region, and years in the profession among the respondents

	**Dentists**	Dental hygienists	Total	
Sent to: [n]	1727	941	2668	
**Participation rate [n (%)]**	241 (13.9)	257 (27.3)	498 (18.6)	
**Age (years) (mean ± S.D)**	40.5 ± 11.3	43.2 ± 12.0	41.9 ± 11.7	
20–29	39 (16.2)	37 (14.4)	76 (15.3)	
30–39	94 (39.0)	78 (30.3)	172 (34.5)	
40–49	54 (22.4)	56 (21.8)	110 (22.1)	
50–59	32 (13.3)	55 (21.4)	87 (17.5)	
60+	22 (9.1)	31 (12.1)	53 (10.6)	
**Sex** [ n (%)]				
Female	173 (71.8)	244 (94.9)	417 (83.7)	
Male	67 (27.8)	10 (3.9)	77 (15.5)	
Don´t wish to state gender	1 (0.4)	3 (1.2)	4 (0.8)	
**Years in profession [ n (%)]**				
< 2 years	22 (9.1)	20 (7.8)	42 (8.4)	
2–5 years	57 (23.7)	37 (14.4)	94 (18.9)	
5–10 years	52 (21.6)	60 (23.3)	112 (22.5)	
More than 10 years	110 (45.6)	140 (54.5)	250 (50.2)	
Stockholm	47 (19.5)	29 (11.3)	76 (15.3)	
Västra Götaland	140 (58.1)	155 (60.3)	295 (59.2)	
Skåne	36 (14.9)	34 (13.2)	70 (14.1)	
Dalarna	9 (3.7)	14 (5.4)	23 (4.6)	
Kalmar	9 (3.7)	25 (9.7)	34 (6.8)	

There was a similar proportion of dentists and dental hygienists among the respondents (dentists: 48.4%, dental hygienists: 51.6%). Most of both dentists and dental hygienists were women (83.8%). The mean age was 42 years (Table 1). A significant proportion of participants (86.3%) had completed their education in Sweden (73.4% dentists and 98.1% dental hygienists). Additionally, 96% stated they had received knowledge about saliva and dry mouth during their undergraduate studies, while 86.9% stated practical training in measuring salivary secretion during their undergraduate education. Only 11.8% reported attending continuing education related to saliva and dry mouth, while 14.9% had received an internal education at their clinic. Respondents reported encountering patients of all ages (3 years − 65 years and older).

### Frequency of patients with oral dryness

Among the respondents, 52.3% stated that they met patients with oral dryness several times per week or more often (Table 2). A higher percentage of dental hygienists (61.1%) than dentists (48.5%) stated that they met individuals with oral dryness several times/weeks or more often (p < 0.01). There was no statistically significant difference in the frequency of meeting individuals with oral dryness based on the length of professional experience (< 10 years or > 10 years), for both dentists and dental hygienists.

**Table 2:** The frequency at which dentists and dental hygienists reported encountering individuals with oral dryness

	Dentists [n (%)]	Dental hygienists [n (%)]	Total [n (%)]
Several times/day	17 (7.1)	18 (7.0)	49 (9.8)
Once/day	9 (3.7)	18 (7.0)	27 (5.4)
Several times/week	84 (34.9)	101 (39.3)	185 (37.1)
Once/week	57 (23.7)	45 (17.5)	102 (20.5)
Once/month	30 (12.4)	26 (10.1)	56 (11.2)
Less than once/month	29 (12.0)	25 (10.1)	54 (10.8)
Never	1 (0.4)	0	1 (0.4)
**Total [n]**	227	257	498

### Assessment of dry mouth

**Fig. 1:**
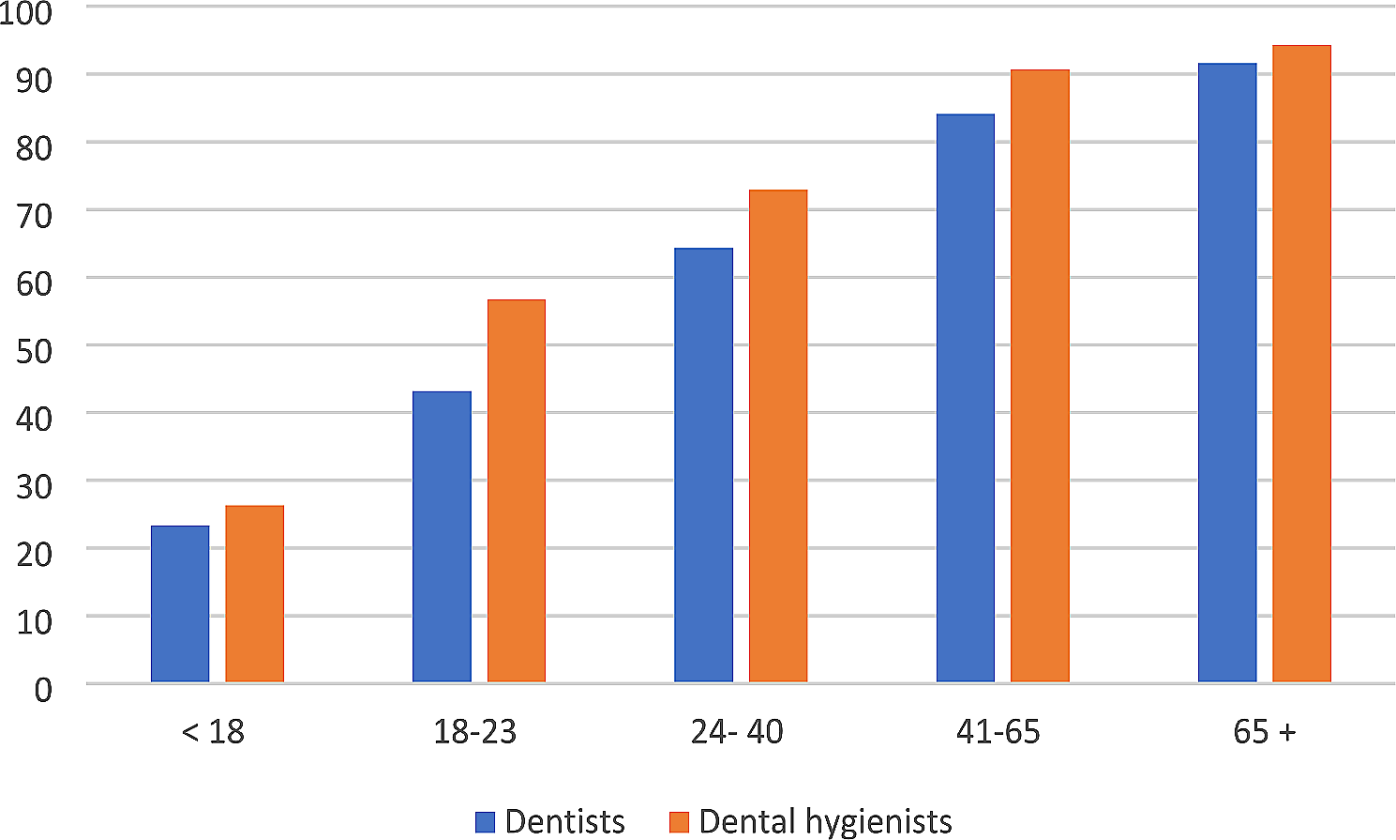
Proportions who inquire about dry mouth in different age groups. Differences between the professions; <18 years (p = 0.455), 18–23 years **(p = 0.003)**, 24–40 years **(p = 0.045)** and 41–65 years **(p = 0.031)**

A total of 93.0% of dental care professionals reported asking individuals aged 65 and older about dry mouth. A lower percentage of dental care professionals, 50.0%, stated that they asked individuals 18–23 years old, and only 24.9% reported asking individuals younger than 18 years. Dental hygienists were found to ask significantly more age groups about dry mouth than dentists, including the age groups 18–23 years, 24–40 years, and 41–65 years (Fig. 1). There were no statistically significant differences regarding the age groups asked about dry mouth and the length of professional experience (< 10 years or > 10 years), for neither dentists nor dental hygienists.

### When is a patient asked about dry mouth?

The most common answer by both professions was that they asked the patient about dry mouth during anamnesis (dentists 88.5% and dental hygienists 90.0%). Additionally, 75.9% of dental hygienists and 74.2% of the dentists asked patients currently on medication, about dry mouth For patients with existing diseases, 60.2% of the dental hygienists and 56.2% of the dentists asked about dry mouth. Significantly more dental hygienists (85.5%) than dentists (78.3%) asked patients about dry mouth based on clinical signs (p = 0.047). Dental hygienists with over 10 years (95.4%) of professional experience were more likely to ask about dry mouth during anamnesis than those with less than 10 years (83.6%) in the profession (p < 0.002).

### Identifying individuals with oral dryness

The respondents were instructed to select three of the eight response options. The three most common indicators to identify individuals with oral dryness were patients’ current and past medication use (92.9% dentists, 88.7% dental hygienists), clinical examination of mucosa and tongue (81.9% dentists, 79.4% dental hygienists), and previous and current diseases (63.7% dentists and 63.2% dental hygienists). There were no statistically significant differences observed neither between the professions nor between dentists/dental hygienists and length of professional experience (< 10 years or > 10 years).

### Determining salivary secretion as a dentist or dental hygienist

A total of 350 (70.3%) stated that they had measured patients’ saliva secretion. Among them, significantly more dental hygienists (88.3%) had performed this measurement, compared to dentists (51.0%) (p < 0.001). Higher proportions of both dentists and dental hygienists with over 10 years of professional experience (69.1% vs. 95.7%) had performed salivary secretion rate determination, compared to those with less than 10 years (35.9% vs. 79.5%) (p < 0.001 for both).

### Indications for measuring unstimulated and stimulated salivary secretion rate

Most dental care professionals stated that Subsidy (special dental care allowance) was the most common reason for measuring the salivary secretion rate, with 76.3% for *unstimulated* saliva and 75.9% for *stimulated* saliva. Clinical signs of dry mouth were the second most common reason for determining the salivary secretion rate (53.1% for unstimulated and 53.3% for stimulated). This was followed by answers to the medical-dental anamnesis (41.0% for unstimulated and 40.6% for stimulated), and upon patients’ request with 30.9% for unstimulated and 31.3% for stimulated, respectively.

Significant differences between the professions were seen, regarding indications for measuring the *stimulated* and unstimulated salivary secretion, as shown in (Table 3). Higher proportions of dental hygienists with less than 10 years in the profession reported measuring the *unstimulated and stimulated* salivary secretion “upon patients request” (43.4% vs. 45.1%), compared to those with over 10 years of professional experience (26.9% vs. 24.6%) (p = 0.007 and p < 0.001).

**Table 3:** Indications for measuring salivary secretion rate and significant differences between the professions

	Unstimulated saliva			Stimulated saliva		
	*Dentists* [%]	*Dental hygienists* [%]	*p- value*	*Dentists* [%]	*Dental hygienists* [%]	*p-value*
Application for subsidy	65.0	86.6	**< 0.001**	64.2	86.6	**< 0.001**
Clinical signs of dry mouth	60.2	46.6	**0.003**	57.5	49.4	0.077
Answers to the medical-dental anamnesis	47.8	34.8	**0.004**	47.8	34.0	**0.002**
Upon patients’ request	27.0	34.4	0.081	28.3	34.0	0.183

### Recall interval for preventive dental care

The most common recall interval set by dental care professionals was twice a year 49.7%, followed by three times a year (19.7%), and four times a year (17.1%). A significantly higher proportion of dental hygienists (42.5%), compared to dentists (30.5%), suggested that patients with oral dryness should receive preventive dental care 3–4 times a year (p < 0.007). A higher proportion of dental hygienists with over 10 years of professional experience (50.7%), compared to those with less than 10 years of professional experience (32.7%), suggested that individuals with oral dryness should receive preventive dental care 3–4 times a year (p < 0.004). No statistically significant difference was seen for recall intervals between dentists and the length of professional experience.

### Providing information about oral dryness

A total of 98.4% responded that they always or often inform patients verbally about oral dryness, 33% always or often provided written information, and 57.4% always or often handed out an information sheet with recommended product/s. A significantly higher proportion of dental hygienists (37.4%), compared to dentists (27.8%), stated that they always or often provided written information (p = 0.031) and handed out an information sheet with recommended product/s (66.7% vs. 46.3%) (p < 0.001). There was no difference between dental hygienists with over 10 years or less than 10 years in the profession. However, dentists with over 10 years of professional experience provided written information to a higher extent than those with less than 10 years (40.9% vs. 17.9%) (p < 0.001).

### Treatment options recommended by dentists and dental hygienists

**Fig. 2:**
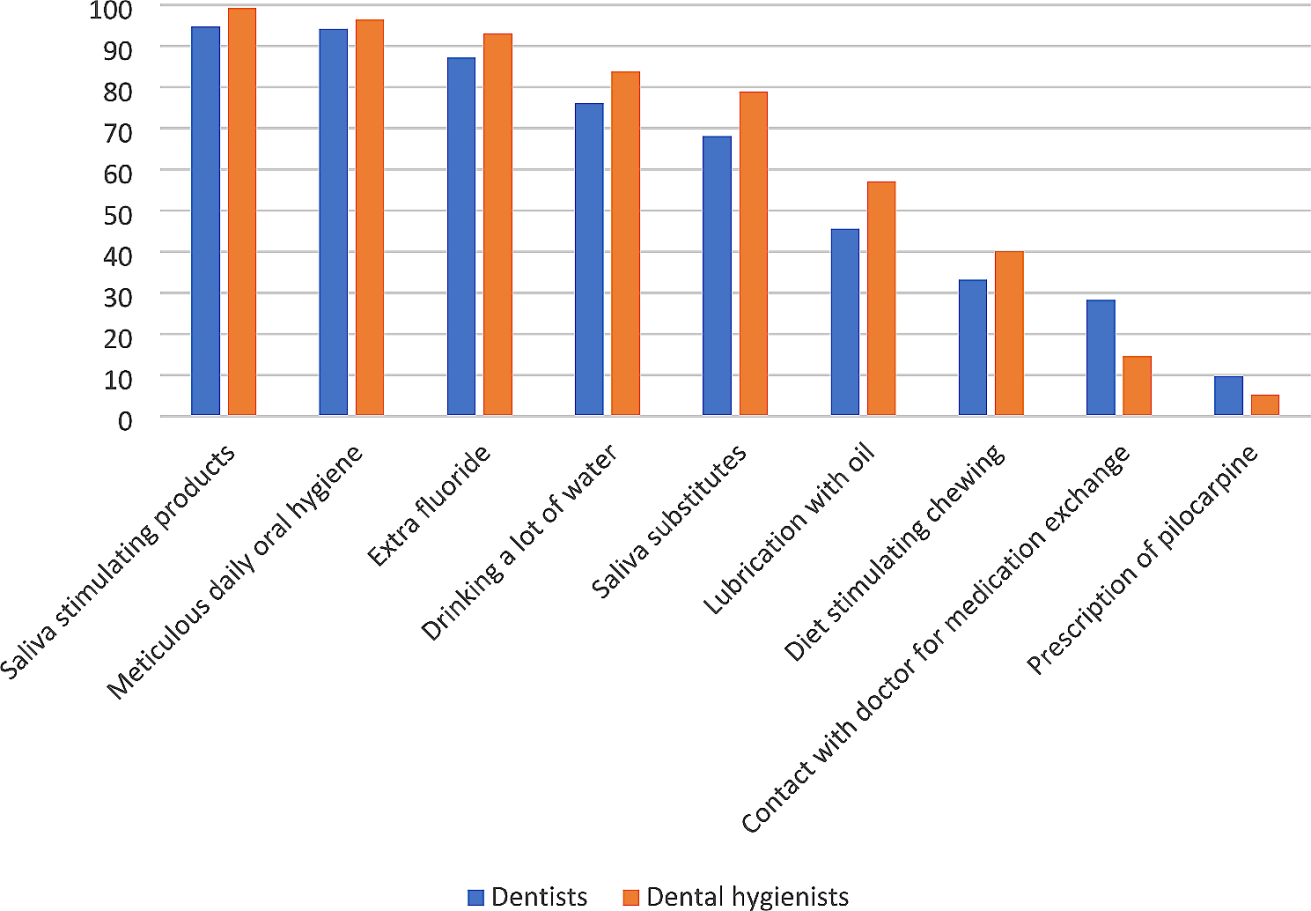
Most (always/often) recommended treatment options provided by dentists and dental hygienists. Differences between the professions and the following treatment options: Saliva stimulating products **(p = 0.004)**, meticulous daily oral hygiene (p = 0.277), extra fluoride **(p = 0.029)**, drinking a lot of water **(p = 0.036)**, saliva substitutes **(p = 0.008)**, lubrication with oil **(p = 0.012)**, diet stimulating chewing (p = 0.120), contact with a doctor for medication exchange **(p < 0.001)**, prescription of pilocarpine **(p = 0.064)**

The three most common recommendations to patients with oral dryness were saliva-stimulating products (97.0%), meticulous oral hygiene (95.3%), and extra fluoride (90.3%). There were differences between the professions in terms of recommended treatment options (Fig. 2). A higher proportion of dental hygienists with over 10 years’ experience, compared to those with less than 10 years in the profession, recommended meticulous oral hygiene (98.5% versus 93.8%) (p = 0.049), a diet that encourages chewing (46.3% versus 32.7%) (p = 0.031), and extra fluoride (97.0% versus 88.5%) (p = 0.008).

### Awareness of the functions of saliva

Between 97.9% and 99.2% of both dentists and dental hygienists were aware with the functions of saliva. There were differences between the professions regarding awareness of saliva functions, as shown in Table 4. However, there were no statistically significant differences between dentists and dental hygienists, or between the lengths of professional experience, in their answers to these questions.

**Table 4:** Number and percentages who strongly agree/agree regarding the functions of saliva, factors which can cause oral dryness and consequences of oral dryness

Question	Dentists	Dental hygienists	p-value
	n (%)	n (%)	
**What are the functions of saliva?**			
Prevents dehydration of oral mucosa	239 (99.2)	255 (99.2)	1.000
Antimicrobial effect	223 (92.5)	216 (84.0)	**0.003**
Regulates pH	236 (97.9)	253 (98.4)	0.745
Affects oral microbiome	223 (92.5)	221 (86.0)	**0.019**
Has lubricating effect	239 (99.2)	256 (99.6)	0.613
Protects oral mucosa	237 (98.3)	255 (99.2)	0.437
Cleanses the oral cavity from food debris	218 (90.5)	**246 (95.7)**	0.020
Facilitates swallowing	238 (98.8)	256 (99.6)	0.358
Facilitates chewing	228 (94.6)	246 (95.7)	0.562
Affects articulation of speech	217 (90.0)	244 (94.9)	0.037
**What can cause oral dryness?**			
Sjögren’s syndrome	240 (99.6)	253 (98.4)	0.374
Parkinson’s disease	133 (55.2)	167 (65.0)	**0.026**
Diabetes Mellitus Type 1	116 (48.1)	149 (58.0)	**0.028**
Diabetes Mellitus Type 2	239 (99.2)	256 (99.6)	0.613
Medication	241 (100)	255 (99.2)	0.5
Radiotherapy head/neck	240 (99.6)	247 (96.1)	**0.008**
HIV/AIDS	84 (34.9)	104 (40.5)	0.197
Cystic Fibrosis	100 (41.5)	111 (43.2)	0.702
Depression and anxiety	187 (77.6)	226 (87.9)	**0.002**
Rheumatic diseases	156 (64.7)	175 (68.1)	0.427
**What complications can oral dryness cause?**			
Increased caries risk	241 (100)	256 (99.6)	1.000
Difficulty speaking	200 (83.0)	240 (93.4)	**< 0.001**
Difficulty swallowing/chewing	228 (94.6)	251 (97.7)	0.075
Increased risk of oral mucosal changes	174 (72.2)	195 (75.9)	0.349
Difficulty in wearing dentures	239 (99.2)	256 (99.6)	0.613
Affects general health	184 (76.3)	215 (83.7)	**0.041**
Quality of life is affected	226 (93.8)	246 (95.7)	0.33
Affects well-being	216 (89.6)	242 (94.2)	0.063
Nutritional deficiency	126 (52.3)	155 (60.3)	0.071
Bad breath	213 (88.4)	242 (94.2)	**0.022**
Altered taste perception	224 (92.9)	235 (91.4)	0.532

### Awareness of factors associated with oral dryness

Dental care professionals were aware that the most common factors causing oral dryness are medications, Sjögren’s syndrome, and radiotherapy to the head and neck (Table 4). Almost all dentists and dental hygienists were aware that Diabetes Mellitus Type 2 can be associated with oral dryness (Table 4). Significantly higher proportions of dentists with over 10 years of professional experience, compared with those with less than 10 years of professional experience, stated that the following factors were associated with oral dryness; rheumatic disease (75.5% compared to 55.7%%) (p = 0.001), and depression and anxiety (84.5% compared to 71.8%) (p = 0.018). Similar results were seen among dental hygienists, where a higher proportion of those with over 10 years of professional experience stated that rheumatic disease (79.3% compared with 54.7%) (p < 0.001), and depression and anxiety (93.6% compared with 81.2%) (p = 0.002), HIV/AIDS (46.4% compared with 33.3%) (p = 0.033), and radiotherapy for head and neck cancer (99.3% compared with 92.3%) (p = 0.004), were associated with oral dryness.

### Awareness of Complications of oral dryness

The majority (99.8%) of the dental care professionals were aware that oral dryness leads to an increased caries risk. The respondents were also aware that oral dryness may lead to complications such as speech difficulties (88.4%), impact general health (80.1%), affect quality of life (94.8%), and well-being (92.0%). Regarding the possibility that oral dryness may lead to nutritional deficiency, 52.3% of dentists and 60.3% of dental hygienists were aware (Table 4). A significantly higher proportion of dental hygienists with over 10 years of professional experience, compared with those with less than 10 years of professional experience, stated that well-being can be affected by oral dryness (97.9% compared with 89.7%) (p = 0.006).

### Self-assessment questions

Overall, dental hygienists rated their knowledge slightly higher than dentists, except regarding the consequences of oral dryness, where the answers were similar (Table 5). Statistically significant differences were shown between the professions in all self-assessment questions (Table 5). Dental hygienists with over 10 years of professional experience were significantly more likely to rate their knowledge higher concerning clinical management (87.1% compared with 75.2%) (p = 0.014), and treatment of individuals with dry mouth (80.7% compared with 69.2%) (p = 0.033), compared to those with less than 10 years of professional experience.

**Table 5:** Responses to self-assessment questions. Numbers and proportions of dentists and dental hygienists [n (%)]

	Very good			Good			Satisfying			Lack of			
	*Dentists*	*Dental hygienists*		*Dentists*		*Dental hygienists*	*Dentists*		*Dental hygienists*	*Dentists*	*Dental hygienists*		
Consequences of oral dryness	49 (20.3)	92 (35.8)		128 (53.1)		123 (47.9)	54 (22.4)		40 (15.6)	10 (4.1)	2 (0.8)		
Clinical management of individuals with oral dryness	25 (10.4)	76 (29.6)		115 (47.7)		134 (52.1)	77 (32.1)		40 (15.6)	24 (10.0)	7 (2.7)		
Treatment of individuals with oral dryness	23 (9.5)	73 (28.4)		105 (43.6)		121 (47.1)	75 (31.1)		54 (20.2)	38 (15.8)	11 (4.3)		
**Dichotomization**													
**Dichotomization very good/good**			*Dentists*		*Dental hygienists*			*p-value*					
Consequences of oral dryness			177 (73.4)		215 (83.7)			**0.005**					
Clinical management of individuals with oral dryness			140 (58.1)		210 (81.7)			**< 0.001**					
Treatment of individuals with oral dryness			128 (53.1)		194 (75.5)			**< 0.001**					

### Request for further education on saliva and oral health

A total of 75.4% of the dentists and 74.1% of the dental hygienists expressed a wish for further education regarding saliva and oral health. A higher proportion of dental hygienists with less than 10 years in the profession, compared to those with over 10 years of professional experience, expressed a wish for further education (83.8% compared with 66.4%) (p = 0.002).

## Discussion

The main results of this exploratory cross-sectional survey study revealed that the majority of dental care professionals (88.6%) inquired about oral dryness in individuals aged 65 and older, while significantly fewer inquiries were made among children, adolescents, and adults. This was seen despite their high exposure to individuals with oral dryness and dental care professionals’ awareness about the functions of saliva and the causes and consequences of oral dryness. Dental hygienists more often asked individuals about dry mouth, including individuals aged 18–65 years, compared to dentists even though both professions met individuals in all age-groups. This may be attributed to the fact that dental hygienists in the General Public Dental Service in Sweden now perform a greater number of oral examinations than in the past. Oral dryness is also prevalent in younger age groups [19], associated with the use of medications for asthma [26], neuropsychiatric diagnoses, and depression, which can increase the risk of oral dryness and accompanying problems [1, 27]. It is important to inquire about oral dryness in all patients, irrespective of age.

According to the results, dental hygienists more often encountered individuals with oral dryness. Preventive dental care for individuals with oral dryness is mainly provided by dental hygienists with frequent follow-ups and information about oral dryness and its associated risks to oral health. The study also shows that dentists with more professional experience provided written information to a higher extent, compared to those with fewer years in the field. Additionally, dental hygienists with over 10 years of professional experience planned more frequent visits for preventive measures and assessed their knowledge higher than those with less years in the profession. These differences could be attributed to the extensive experience of these dental care professionals in examining and treating persons with oral dryness and witnessing deteriorating oral health, thereby understanding the need for preventive and supportive oral health care. Another explanation could be that dentistry in Sweden has changed over the years. Factors such as a growing shortage of dental care professionals, and a greater demand for aesthetic dentistry, may have resulted in oral dryness being less prioritized. Studies have shown that there is a general lack of interest in prevention among dentists, where dentists perceived that prevention has a lower priority than treatment and is not part of their daily practice [28, 29]. It has also been shown that dentists consider preventive work to be a part of the dental hygienists’ job task [29, 30]. Most of the dental care professionals (81.4%) who did not answer the questionnaire might have a low interest in preventive dentistry. Preventive dentistry should be encouraged among dental care professionals during their clinical practice.

In most regions in Sweden, it is common to include one yes or no question about a dry mouth in the medical-dental anamnesis. However, it could be doubted if this gives sufficient knowledge about the severity of a patient’s oral dryness problems. It is possible that dental care professionals ask follow-up questions regarding oral dryness in various situations, but such questions were not included in the questionnaire used in this study. Existing literature suggests the use of validated questionnaires to assess the severity of oral dryness. One such questionnaire is the Xerostomia Inventory (XI), used in the assessment of chronic xerostomia severity [31]. A complete medical-dental history and diagnostic tests, e.g., salivary flow measurement, are appropriate patient assessments when diagnosing xerostomia or hyposalivation, to identify the underlying cause [32], as shown in the current study. However, it should be noted that the results did not provide information regarding specific diagnosis assessment between xerostomia and hyposalivation. The Clinical Oral Dryness Scale (CODS) is recommended in the literature to evaluate clinical signs of of oral dryness and grade its severity [33], though the Xerostomia Inventory or CODS is not used in daily practise by dental care professionals in Sweden.

Patients diagnosed with hyposalivation (as opposed to xerostomia) in Sweden are eligible to receive a special dental care allowance because of the increased risk of deteriorating oral health. For a diagnosis, both the unstimulated and the stimulated saliva secretion rate are measured. Individuals showing hyposalivation (indicated by an unstimulated saliva flow of ≤ 0.1 ml/min, and stimulated saliva flow of ≤ 0.7 ml/min) become eligible for the allowance and can receive 1200 SEK/year to be used for check-ups and preventive oral care [34, 35]. In the present study, the dental care allowance was the main indicator for measuring unstimulated and stimulated salivary secretion by both professions. It was performed by both dental hygienists and dentists, however, a significantly higher proportion of dental hygienists reported encountering patients with oral dryness more frequently compared to dentists.

Dry mouth is often a chronic condition. To relieve symptoms of dry mouth and decrease the risk of oral health problems, saliva-stimulating products (lozenges, chewing-gum and sprays) and saliva substitutes (gel, spray and oil) are used [36]. However, a Cochrane review shows no firm evidence for using topical therapies to relieve symptoms of dry mouth [37]. The present study shows that the three most recommended treatment options suggested by dental care professionals were stimulating products (97.0%), meticulous oral hygiene (95.3%), and extra fluoride (90.3%). While meticulous oral hygiene with fluoridated toothpaste is important in reducing the risk of both caries and periodontal diseases [38, 39], and extra fluoride in rinses or gels is recommended to reduce the risk of dental caries [40], neither options can effectively relieve dry mouth. The proportion of dental care professionals in the present study recommending extra fluoride was higher compared to a study from England, where only 10% of dentists prescribed fluoride rinse [23]. The explanation may be attributed to recommendations from the Swedish National Guidelines for Dental Care, where individuals with increased risk of caries should receive extra fluoride such as 0.2% sodium fluoride as a rinse, 0.42% fluoride gel in trays, fluoride toothpaste with 5000 ppm sodium fluoride, or fluoride varnish (22.6 mg/ml) at least twice a year [41, 42]. In the present study, 70–80% of the dentists and dental hygienists recommended frequent water intake for patients with dry mouth, which is in concordance with a German study [22]. However, patients with dry mouth report that water only provides temporary relief from their symptoms [15]. Water is inexpensive, easy to carry, and readily available, with no negative side-effects, which likely explains why it is a common recommendation.

This study shows that only 33% of dental care professionals provided written information about dry mouth, with 57.4% stating that they frequently distributed an information sheet with recommended products. A recent report has highlighted that patients have limited recall of verbal instructions provided during their dental visit, suggesting a risk of dental care professionals underestimating the patients’ need for written information [43]. A literature review has shown that patients request digital, as well as oral and written information [44]. This study shows no statistically significant difference regarding the length of professional experience among dental hygienists and ways of providing information regarding oral dryness to patients. This could be attributed to the fact that providing information has always been an integral part of a dental hygienist’s role, regardless of when they completed their education.

The dental care professionals participating in this study were aware of the complications dry mouth can cause. Both dentists (93.8%) and dental hygienists (95.7%) acknowledged that quality of life can be affected when experiencing dry mouth, which is in congruence with a previous study [23]. Similarly, awareness regarding the impact of dry mouth on general health was high in this study and in congruence with previous results [23]. A high proportion (75.9%) of the dental care professionals stated that they never or seldom consult with physicians to change medications because of adverse effects such as dry mouth. This may be attributed to the limited interprofessional collaboration between Public Dental Care and medical healthcare services, which impacts on the treatment options and preferences of dental professionals. Research conducted in other settings aiming to include dental care professionals in interprofessional teams has shown positive results with beneficial outcomes for patients’ oral health [45].

### Strengths and limitations

The strengths of this study were the similar numbers of dentists and dental hygienists answering the questionnaire, facilitating comparisons between the groups. The survey was sent to five of the 21 regions in Sweden where the three largest cities, as well as rural areas, were included. Therefore, it can be argued that the included regions represent the whole of Sweden. The use of questionnaires as a research tool is well-documented and widely used in various settings. Online questionnaires are suitable when conducting a cross-sectional study, as respondents are contacted only once [46]. The authors of this study developed the questionnaire, which may have limitations in terms of validity and reliability. To the best of our knowledge, there are no validated questionnaires available on this topic. Only Public Dental Care organizations agreed to take part in this study, despite several Private Dental Care organizations being invited to participate. This might affect the results, as 60% of adults are treated in Private Dental Care clinics [47].

The response rate in the present study was low (18.6%), but comparable to an earlier study in the same field [22]. The low response rate might be attributed to lack of interest in oral dryness or high workload, because of a shortage of dentists and dental hygienists in most regions of Sweden [48], leaving limited time for tasks other than direct patient care. Oral dryness should be interesting for dental care professionals since they frequently meet patients with this condition. It is likely that respondents in the present study are those who are more interested in oral dryness or dental care of this patient group, which may be a potential bias. Efforts were made to minimize non-response, such as sending more than one reminder and the respondents could choose to re-open and continue to fill in the questionnaire at another time-point. Information regarding the characteristics of respondents and non-respondents was not available, therefore a non-response analysis was impossible. However, according to statistics provided by the Regions and by the Swedish National Board of Health and Welfare [49], similarities existed between the dental care professionals included in this study and all licensed dental care professionals in the respective Regions and the whole of Sweden, such as gender and age. The results should be interpreted with caution, since results cannot be generalized. Despite limitations, the results are valuable due to the lack of studies on managing oral dryness in general dental care in Sweden.

### Future perspectives

It is important to improve the diagnosis of individuals with oral dryness regardless of age to provide preventive measures to reduce the risk of oral diseases. It is advisable to enrich the educational curriculum for dentist and dental hygienist students by incorporating not only a deeper understanding of pharmacology but also by providing continuing education focused on saliva and the management of oral dryness. In the future, it is important to include Private Dental clinics in the research, as they see larger proportions of individuals with dry mouth problems. Currently, a qualitative study is ongoing, involving interviews with dentists and dental hygienists in both the private and Public Dental Care sectors about their experiences with oral health care for individuals with oral dryness.

## Conclusions

Compared to dentists, dental hygienists were more attentive to patients with oral dryness as they encountered these individuals more often, asked more age-groups, suggested frequent preventive measures, and had higher awareness of the causes and complications of oral dryness. Length of professional experience could improve both the management of patients with oral dryness and awareness of its causes, particularly for dental hygienists.

## Data Availability

The datasets utilized and/or analysed during the current study can be obtained from the corresponding author upon reasonable request.
